# Trends in antibiotics resistance for urinary tract infections - A five-year retrospective study in India

**DOI:** 10.6026/973206300213010

**Published:** 2025-09-30

**Authors:** Balvir Singh, Archana Patel, Ramesh Prasad Agrawal, Sudhir Kumar Jain

**Affiliations:** 1 Department of Pharmacology, Government medical College, Satna, Madhya Pradesh, India; 2 Department of Pharmacology, Smt. B.K. Shah Medical Institute and Research Centre, Sumandeep Vidyapeeth, Waghodia, Vadodara, Gujarat, India; 3 Department of Microbiology, Government medical College, Satna, Madhya Pradesh, India; 4 Department of Pharmacology, Atal Bihari Vajpayee Government Medical College, Vidisha, Madhya Pradesh, India

**Keywords:** Urinary tract infection, antibiotic resistance, *escherichia coli*, multidrug resistance, retrospective study

## Abstract

Analysis of 135 culture-positive urinary tract infection (UTI) cases at a tertiary care center to evaluate trends in antibiotic resistance is of interest. *escherichia coli* remained the most prevalent uropathogen, followed by *Klebsiella* and *Pseudomonas species*. Resistance to commonly used antibiotics like ciprofloxacin and ampicillin increased significantly over the years, while sensitivity to nitrofurantoin and fosfomycin remained relatively preserved. A worrying rise in multidrug-resistant (MDR) strains was noted, particularly among hospitalized patients. Thus, the need for region-specific antibiotic stewardship to curb escalating resistance patterns is shown.

## Background:

Urinary tract infections (UTIs) are among the most common bacterial infections encountered in both community and hospital settings, affecting individuals of all ages and genders [1]. *escherichia coli* are the predominant causative organism, followed by other Gram-negative bacteria like *Klebsiella*, *Proteus and Pseudomonas* [2]. Empirical antibiotic therapy is often initiated before culture results, which makes continuous monitoring of antimicrobial resistance patterns vital for effective treatment [3]. In recent years, increasing antibiotic resistance has become a global health concern, with UTIs showing an alarming rise in resistance to frequently prescribed antibiotics such as fluoroquinolones, cephalosporins and beta-lactam antibiotics [4]. The emergence of multidrug-resistant (MDR) uropathogens has led to therapeutic challenges, higher healthcare costs, prolonged hospital stays and increased morbidity [5]. Resistance trends vary geographically and temporally, necessitating local surveillance data to guide empirical therapy [6]. Therefore, it is of interest to assess the trends in antibiotic resistance patterns of uropathogens isolated from UTI patients over a five-year period. By identifying evolving resistance profiles, the study seeks to inform clinical decision-making and promote rational use of antibiotics through evidence-based antimicrobial stewardship.

## Materials and Methods:

This retrospective study was conducted at the Microbiology Department of a tertiary care hospital, analyzing urine culture and sensitivity reports from January 2019 to December 2023. A total of 135 non-repetitive, culture-positive urinary tract infection (UTI) cases were included. Patients of all ages and both sexes were considered, provided they had clinically diagnosed UTIs and significant bacteriuria (≥10^5^ CFU/mL) on culture. Only the first isolate from each patient was included to avoid duplication. Patients who had received antibiotics within 48 hours before sample collection or had polymicrobial infections were excluded. Urine samples were collected using the clean-catch midstream technique or catheter aspiration in admitted patients. Samples were cultured on MacConkey and blood agar plates and incubated at 37°C for 24 hours. Identification of organisms was done using standard biochemical tests. Antibiotic susceptibility testing was performed using the Kirby-Bauer disk diffusion method in accordance with CLSI guidelines. Antibiotics tested included ampicillin, ciprofloxacin, ceftriaxone, ceftazidime, nitrofurantoin, fosfomycin, piperacillin-tazobactam and imipenem. The data were categorized year-wise and analyzed for resistance trends. Multidrug resistance (MDR) was defined as resistance to at least one antibiotic in three or more antimicrobial categories. Results were entered and analyzed using SPSS version 26. Descriptive statistics were used for frequencies and proportions and Chi-square test was applied to detect significant changes in resistance patterns over time. A p-value <0.05 was considered statistically significant.

## Results:

A total of 135 culture-positive urinary tract infection cases were analyzed over a five-year period (2019-2023). The most commonly isolated organism was *escherichia coli*, followed by *Klebsiella pneumoniae* and *Pseudomonas aeruginosa*. A significant increase in resistance was observed for fluoroquinolones and third-generation cephalosporins, while nitrofurantoin and fosfomycin retained higher sensitivity rates. Multidrug-resistant (MDR) strains showed a rising trend, especially in hospital-acquired infections. Table 1 (see PDF) shows *escherichia coli* remained the predominant pathogen over all five years, followed by *Klebsiella* and *Pseudomonas*. Table 2 (see PDF) shows Resistance to ciprofloxacin increased significantly among *E. coli* isolates, indicating declining efficacy. Table 3 (see PDF) shows Nitrofurantoin remained relatively effective against *E. coli*, with only a slight rise in resistance. Table 4 (see PDF) shows Ampicillin resistance remained consistently high among *E. coli* isolates. Table 5 (see PDF) shows Fosfomycin remained highly effective against *E. coli*, showing minimal resistance over the years. Table 6 (see PDF) shows Resistance among *Klebsiella* isolates to third-generation cephalosporins rose sharply, indicating possible ESBL production. Table 7 (see PDF) shows Imipenem retained effectiveness in all years, showing no resistance among major isolates. Table 8 (see PDF) shows the proportion of multidrug-resistant (MDR) strains increased gradually over the five years. Table 9 (see PDF) shows Hospital-acquired UTIs had significantly higher MDR prevalence compared to community-acquired infections. Table 10 (see PDF) shows Male patients showed a slightly higher resistance pattern, especially in hospital settings.

## Discussion:

This five-year retrospective study reveals important trends in antibiotic resistance among urinary tract infection (UTI) pathogens in a tertiary care setting. *escherichia coli* remained the most prevalent uropathogen, consistent with global data, followed by *Klebsiella pneumoniae* and *Pseudomonas aeruginosa*. The resistance patterns observed indicate a disturbing rise in resistance to commonly prescribed antibiotics, particularly ciprofloxacin, ampicillin and third-generation cephalosporins [7]. The increasing resistance to ciprofloxacin among *E. coli* from 52.9% in 2019 to 84.6% in 2023 reflects the overuse and empirical misuse of fluoroquinolones. Similarly, ampicillin resistance remained consistently high throughout the study period [8]. On the other hand, nitrofurantoin and fosfomycin continued to be relatively effective against *E. coli*, although a slow upward trend in resistance suggests the beginning of possible future challenges. These drugs could remain valuable options for empirical treatment of uncomplicated UTIs, especially in outpatient settings [9]. *K. pneumoniae* showed alarming levels of resistance to ceftriaxone in later years (100% resistance in 2022 and 2023), indicating probable widespread presence of ESBL (Extended Spectrum Beta-Lactamase) producing strains [10]. However, the preserved sensitivity of major uropathogens to imipenem suggests that carbapenems remain a last-line effective option, though the emergence of resistance in *Pseudomonas* warrants cautions [11].

The rise in multidrug-resistant (MDR) strains from 23.1% in 2019 to 50% in 2023 is highly concerning and mirrors global antimicrobial resistance (AMR) trends [12]. MDR prevalence was significantly higher in hospital-acquired infections and among male patients, possibly due to more frequent catheterization, comorbidities and prolonged antibiotic exposure in inpatient care [13]. These findings highlight the critical need for hospital-based antimicrobial stewardship programs and stricter infection control practices [14, 15]. Exploring the effects of combined interventions, such as antibiotic education and enhanced collaboration between outpatient and inpatient departments, may offer comprehensive approaches to combating antibiotic resistance [16]. The data underscore a shifting landscape of antibiotic efficacy in UTIs, with traditional first-line agents becoming increasingly ineffective. These resistance trends emphasize the necessity for continuous regional surveillance, judicious antibiotic use and tailored empirical therapy to mitigate the growing burden of antibiotic resistance in urinary infections.

## Conclusion:

A significant upward trend in antibiotic resistance among UTI pathogens, particularly to ciprofloxacin, ampicillin and third-generation cephalosporins is shown. Despite rising resistance, nitrofurantoin, fosfomycin and imipenem remained largely effective. The increasing prevalence of multidrug-resistant strains, especially in hospital-acquired infections, underscores the urgent need for robust antimicrobial stewardship and regular surveillance to guide effective empirical therapy.
